# Contributions and Challenges of High Throughput qPCR for Determining Antimicrobial Resistance in the Environment: A Critical Review

**DOI:** 10.3390/molecules24010163

**Published:** 2019-01-03

**Authors:** Hassan Waseem, Sana Jameel, Jafar Ali, Hamza Saleem Ur Rehman, Isfahan Tauseef, Uzma Farooq, Asif Jamal, Muhammad Ishtiaq Ali

**Affiliations:** 1 Department of Environmental Engineering, Michigan State University, East Lansing, MI 48823, USA; waseemh1@msu.edu; 2 Department of Biotechnology, University of Sialkot, Punjab 51310, Pakistan; jameels@msu.edu (S.J.); h.s.rehman15@gmail.com (H.S.U.R.); 3 Environmental Microbiology Laboratory, Department of Microbiology, Quaid-i-Azam University, Islamabad 45320, Pakistan; asifjamall@yahoo.com; 4 key Laboratory of Environmental Nanotechnology and Health Effects, Research Center for Eco-Environmental Sciences, Chinese Academy of Sciences, 18 Shuangqing Road, Beijing 100085, China; Jafarali_st@rcees.ac.cn; 5 Department of Microbiology, University of Hazara, Mansehra 21300, Pakistan; isfahantauseef@yahoo.com; 6 Department of Plant Sciences, Quaid-i-Azam University, Islamabad 45320, Pakistan; uzma.ishi@gmail.com

**Keywords:** AMR, high throughput qPCR, ARGs, MGEs, gut microbiome

## Abstract

Expansion in whole genome sequencing and subsequent increase in antibiotic resistance targets have paved the way of high throughput qPCR (HT-qPCR) for analyzing hundreds of antimicrobial resistance genes (ARGs) in a single run. A meta-analysis of 51 selected studies is performed to evaluate ARGs abundance trends over the last 7 years. WaferGen^TM^ SmartChip is found to be the most widely used HT-qPCR platform among others for evaluating ARGs. Up till now around 1000 environmental samples (excluding biological replicates) from different parts of the world have been analyzed on HT-qPCR. Calculated detection frequency and normalized ARGs abundance (ARGs/16S rRNA gene) reported in gut microbiome studies have shown a trend of low ARGs as compared to other environmental matrices. Disparities in the HT-qPCR data analysis which are causing difficulties to researchers in precise interpretation of results have been highlighted and a possible way forward for resolving them is also suggested. The potential of other amplification technologies and point of care or field deployable devices for analyzing ARGs have also been discussed in the review. Our review has focused on updated information regarding the role, current status and future perspectives of HT-qPCR in the field of antimicrobial resistance.

## 1. Introduction

Antimicrobial resistance (AMR) is a major public health safety issue and is not a new phenomenon in health and agricultural settings [1,2]. Recently AMR has gained more attention due to ever increasing discharge of pollutants (including antibiotics) into environmental matrices [3,4,5]. Detection of bacterial resistance by conventional methods is time consuming and laborious. Molecular techniques can detect antimicrobial resistance genes (ARGs) in a rapid and sensitive manner. ARGs encode the ability in bacteria to resist and grow in the presence of antibiotics. Although, ARGs presence inside bacteria pose a serious threat but their existence in environmental matrices is of equal concern because environmental bacteria and/or clinical pathogens can acquire ARGs via horizontal gene transfer [6,7]. ARGs have been regarded as environmental pollutants in the past [8]. Previously scientists were only targeting a small fraction of ARGs in environmental matrices [9,10] but with the reduction in the price of sequencing and consequent expansion in whole genomic sequencing of bacteria [11], the number of available ARG targets in different databases have reached to thousands (Table S1). A comprehensive profiling of ARGs by conventional qPCR is time consuming, cost ineffective and burdensome. High throughput qPCR (HT-qPCR) is a relatively rapid and convenient method for the simultaneous evaluation of a large number of ARGs.

Comprehensive analysis of different ARGs is essential to decipher the intricate relationship between ARGs, MGEs and other complex and overlapping environmental factors. For example Sun and co-workers have studied effect of temperature on the ARGs during anaerobic digestion of dairy manure [12]. Similarly assessment of public health safety risks associated with exposure to antimicrobials, ARGs and/or antibiotic resistant bacteria is another important factor to fully understand this area. Insights into the role of different environmental matrices in the dissemination and/or development of antibiotic resistance is also pushing the scientific community towards high throughput profiling of resistance genes [13,14].

Application of manure and its impact on soils is of particular interest in agricultural settings, because soil microbiomes have been known to contain a diverse pool of ARGs, including the most common types of resistances found in other environmental matrices [15,16,17]. Presence of ARGs, without any antimicrobial exposure or treatment, in various animals and human beings is not surprising and has already been observed. For example transmission of microbial communities and associated ARGs between healthy mother-infant pairs [18], where resistance genes were detected in the mouth of new-born immediately after birth [19]. Similarly the presence of ARGs in remote pristine environments has also been reported [20]. Moreover numerous studies have evaluated the ARGs via HT-qPCR in aquatic environments indicating easy transport of ARGs and obvious association of pollution sources in rivers and estuaries [21].

Although various research articles have been published about the detection and prevalence of ARGs in various environmental matrices all over the world, there has not been any review article highlighting the role of HT-qPCR for analyzing the antimicrobial resistance genes. Therefore, the aim of our review is to provide a snapshot of the contributions made by HT-qPCR technology with the perspective of antimicrobial resistance. The specific objectives of the study are as follows: (i) to evaluate and compare HT-qPCR technology with other competitive molecular methods, (ii) to identify the global trends of antimicrobial resistance by meta-analysis of the included studies and (iii) to examine and highlight major discrepancies and disparities in the data analysis of HT-qPCR. Standardized reporting of ARGs abundance in the literature is also suggested. In the last section the potential and a path forward for future applications of new and emerging methods for evaluating ARGs have been discussed.

## 2. Methodology and Meta-Analysis

**Figure 1:**
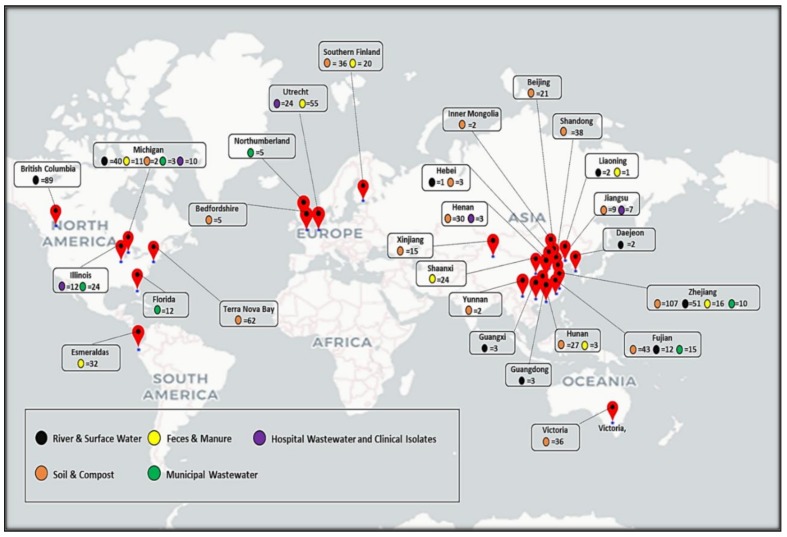
World map showing no. of samples of different environmental matrices analyzed by HT-qPCR over last 7 years in different regions of the world.

For analyzing the role of HT-qPCR in the field of antimicrobial resistance, we have used the Web of Knowledge database (http://apps.webofknowledge.com.proxy2.cl.msu.edu) to collect publications. Our search terms included (high throughput qPCR AND antibiotic resistance). Only studies published up to October, 2018 were included for the analysis. Research articles were then checked manually to remove any duplicates and/or irrelevant articles. Articles which are not published in English language were not considered for the present study. Eventually 51 research articles reporting the use of HT-qPCR for analyzing ARGs met our criteria and were selected for the meta-analysis. Approximately 1000 samples of different environmental matrices, analyzed in 51 included studies, were from different regions of China, United States of America, Finland, Netherlands, UK, Australia, Canada, Antarctica and Ecuador (Figure 1). In particular, 14 published reports evaluated ARGs and MGEs in soil samples from different agricultural sites and farms. Many studies have evaluated effects of chemicals, manure and composts on the abundance of ARGs in soils. Aquatic matrices seems to receive more attention (20/51) than soil related studies.

The number of samples, primers, unique genes detected and normalized gene abundances (ARGs/16S) were provided either in the main text or have been retrieved from figures and/or supplementary material (Table S2). For some studies the ARGs were normalized per bacterial cells. In such cases the values have been multiplied to 4.1 in order to convert the abundances per 16S rRNA gene assuming 4.1 16S rRNA genes were present in one bacteria [22,23]. Region based distribution of samples belonging to different environmental matrices were evaluated and depicted by a world map. World map used in this review was made with the help of geo-coordinate module of heatmapper software [24]. Basic data calculations, bar graphs and pie-chart was made in Microsoft Excel 2010. Box and whisker plots for comparing the normalized ARGs abundances in different matrices were made using Paleontological Statistics Software Package for Education and Data Analysis (PAST: v3.20) [25]. Additionally the number of reference sequences in different resistance associated databases were also retrieved from the published manuscripts and/or websites of the databases (Table S1).

## 3. Competitor Technologies of HT-qPCR for High Throughput Profiling of ARGs

Tremendous amount of work has been published regarding the use of conventional hybridization array technology in the field of antimicrobial resistance [26,27,28,29]. The major advantage of microarray technology over HT-qPCR is the number of ARGs (~1000′s) which can be profiled in one run. However, microarray suffer batch to batch variability and generally considered as relatively less sensitive and specific. This is further exemplified by the fact that microarray data needs additional validation by qPCR [30,31] however, HT-qPCR data does not need additional validations.

Similarly large number of studies are using metagenomic sequencing approach for evaluating the status of ARGs in bacterial communities [32,33,34,35]. The major advantage of metagenomic profiling is its ability to analyze unknown sequences whereas with HT-qPCR it is necessary to know the sequences beforehand for designing the primers. HT-qPCR, on the other hand, certainly provides better detection limits than by metagenomic sequencing approach. For example HT-qPCR has reportedly detected ARGs to the order of magnitude of 10^−4^ ARGs/16S rRNA gene [36,37]. Average bacterial genome size is ~3.67 to ~5.56 million bases [38] and the size of 16S rRNA gene is ~1500 bp [39]. Assuming 10,000 reads are needed to detect a single copy of 16S rRNA gene, the same detection limit for any ARG as shown by HT-qPCR would require at least 10^8^ reads during metagenomic analysis. In addition to high detection limits, the learning curve during the data analysis of HT-qPCR is less steep as it does not involve complex bioinformatics tools and pipelines, a necessity in case of short gun metagenomic approach.

Both metagenomic sequencing and microarray hybridization generally requires more sample and/or DNA quantity. In some instances, scientists have solved this problem by whole genome amplification but this can have impacts on the quality of the final results [40,41]. HT-qPCR has countered this limitation because it can perform reactions in nanoliter scale thus consuming only a minute amount of DNA. The development of HT-qPCR platforms has indeed changed the perception about conventional qPCR and its limitless utilities. This techniques has proved to be very cost-effective as nanoliter scale reactions allow substantial savings in terms of consumables and reagents and also allows a more efficient use of the available sample. All these features along with previous strongholds of conventional qPCR (sensitivity and specificity) have made HT-qPCR a technique of better choice in the context of AMR detection.

HT-qPCR technology has some disadvantages as well. For example, inability of HT-qPCR to optimize individual assays during a run as all assays would experience same qPCR cycling conditions. This can be critical because specific binding of primers often require different annealing temperatures [42,43]. Also the reactions at nanoliter scale make it difficult to recover and sequence amplified products which is easily possible in conventional PCR.

### Different HT-qPCR Platforms Used for ARGs Analysis

**Figure 2:**
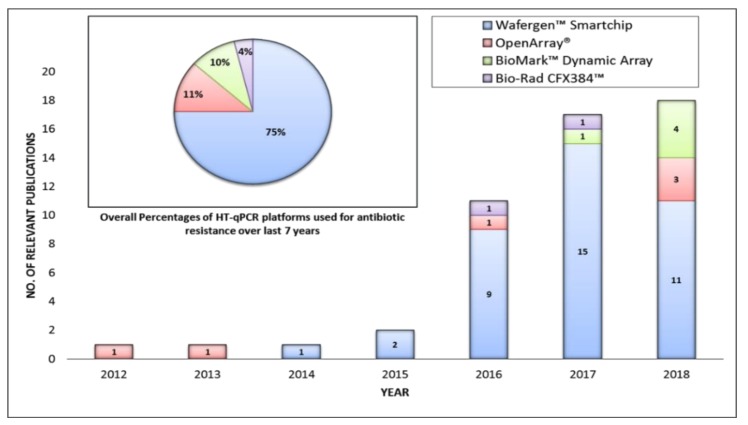
Number of publications using different HT-qPCR platforms over last 7 years.

Currently four different kind of HT-qPCR platforms have been used for profiling of ARGs. These platforms have different high throughput capacities and also uses different reaction volumes (Figure 2). All these platforms have been used for measuring the diversity and abundance of ARGs in different environmental and clinical samples but WaferGen’s platform is the most frequently used platform for analysis and profiling of ARGs. In 75% of the studies included in our analysis, WaferGen^TM^ Smartchip has been used for analysis of antibiotic resistance (Figure 2).

**Figure 3:**
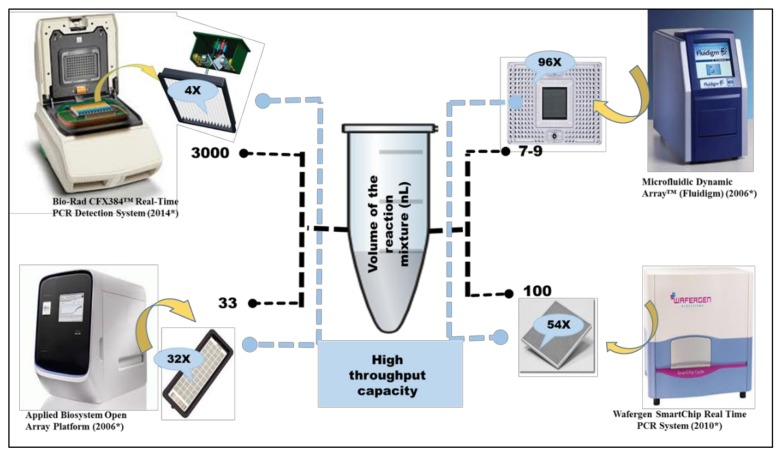
Reaction Volume and High throughput capacity of various HT-qPCR platforms used for ARGs analysis.

Size of the reaction volume can effect qPCR results [44]. Bio-Rad CFX384^TM^ platform provides better analytical sensitivity due to its larger reaction volumes but because of a relatively smaller number of reaction wells (384), it has been employed only in two ARGs related studies for high throughput profiling of the ARGs [45,46]. On the other hand very high throughput capacity (96×) of Biomark^TM^ Dynamic Array (Fluidigm, California, CA, USA) have forced the reduction of reaction volumes to 7–9 nL which can negatively affect its analytical sensitivity. Also the required pre-amplification step [47,48] can also effect the accuracy of the analysis. Open Array^®^ of Applied Biosystems^TM^ allows a higher reaction volume (33 nL) with a reasonable throughput capacity (32×) but the primers in this case has been pre-dispensed by the company causing inflexibility in terms of run to run customization (Figure 3).

WaferGen^TM^ Smartchip provides customized no. of samples versus assays formats which can be opted according to the requirements of the experimental design. Most of the studies have used 296 versus 18 and 384 versus 12 Assay-Sample format. It also has better analytical sensitivity as compared to other microfluidic chips because of larger 100 nL reaction volume. It is perhaps due to these features that scientists are preferring the use of WaferGen^TM^ Smartchip over others HT-qPCR platforms especially in the field of antimicrobial resistance.

## 4. Role and Impact of Community Dynamics and MGEs in ARGs Dissemination

Changes in resistome of bacterial population in response to shifts in environmental parameters such as temperature, pH and so forth, can occur either through vertical gene transfer (selective enrichment of a bacterial population) [49,50] or via horizontal gene transfer of ARGs [51]. Increase in abundance of ARGs along with changes in bacterial community composition and subsequent enrichment of any particular bacterial group have been widely reported. For example *Enterobacteriaceae* increase was positively correlated with ARGs in lakes in Brazilian Zoo [52]. Also during high throughput profiling of ARGs in East Tiaoxi river, role of bacterial community was found to be more effective as compared to mobile genetic elements in influencing the resistome [53]. Similarly, Xiang and colleagues, while studying the spatial and temporal distribution of antibiotic resistomes indicated the variations in bacterial community structure was major contributing factor in the alterations of bacterial resistomes [54]. However, this does not diminish the importance of horizontal gene transfer, an important pathway for dissemination of ARGs, in the development and emergence of multidrug resistant bacteria in different clinical and environmental matrices [55]. For example sub-lethal stress caused by environmental fluctuations such as pH downshifts is reported to accelerate the net rate of inter- and intra-specific transmission of selected antimicrobial resistance plasmids [56]. Jacquiod and colleagues have assessed the conjugative plasmid permissiveness of wastewater microbiomes and found that *Gammaproteobacteria* were preferred hosts highlighting the role of plasmid in mobility of ARGs via horizontal gene transfer [57].

In silico and experimental analysis of MGEs was performed in several studies in order to understand their role in transportation and/or enrichment of ARGs in different environmental matrices [58,59]. *Int*1 gene is known as a major player in the transport of ARGs both within and between different bacterial species. Several molecular assays involving *int*1 gene has already been developed to get an overall view of the ARGs as they are believed to be a proxy of anthropogenic pollution and ARGs [60,61]. Many studies have implied that the co-occurrence of MGEs especially *int*1 can play a negative role by disseminating ARGs in clinically important strains, thus can produce multiple antibiotic resistance bacterial strains which can cause serious public health and safety concerns [62,63,64].

Except few studies included in our analysis, most studies analyzing ARGs have also evaluated the status of MGEs. A total of 9 MGEs including *int*1 were present in 296 primer array. This array has been used in 68.6% (35/51) of the studies included in the review. The number of MGEs was increased to 61 in 384 primer array that has been utilized in various studies [36,65]. In more than half of the studies (26/51), researchers have also analyzed the community structure by sequencing the hypervariable regions of 16S rRNA gene. Different hypervariable regions were amplified and sequenced in different studies, which can affect and/or influence the results of community analysis [66,67]. We will not be discussing the impact and selection criteria for amplification and sequencing of hypervariable regions of 16S rRNA gene as it is out of the scope of the review.

In (27/51) studies researchers have reported significant linear correlation between certain ARGs and MGEs including *int*1. However correlation of ARGs with MGEs or *int*1 is not evident enough to infer that horizontal gene transfer is responsible for enrichment of ARGs. As integrons allow acquisition and dissemination of ARGs within gene cassettes [59,68]. Possibility of an altogether increase of ARGs and MGEs, if the cell carrying the particular plasmid has been positively selected, is always there. In that case microbial community dynamics would be the major driver for enriching the ARGs. Inclusion of only a limited set of MGEs primers in ARGs qPCR array and also the absence of plasmid specific genes is the major limitation of the MGEs analysis via HT-qPCR which needs to be rectified. More in depth analysis with direct and conclusive experimental designs of validatory nature are needed to determine the route of ARGs enrichment in different studies. Studies for establishing linkages between ARGs and MGEs with certainty have focused on procuring long read sequencing, better ways of sequence assembly and also on source tracking methods based on whole genome sequencing of isolates [11]. For example, MinION can provide a read length of 5000 bp and it has been used to evaluate the genomic regions containing all ARGs, MGEs and virulence genes of *Salmonella enterica* serovar Typhi H58 [69].

## 5. Trends of ARGs Diversity in Different Matrices

**Figure 4:**
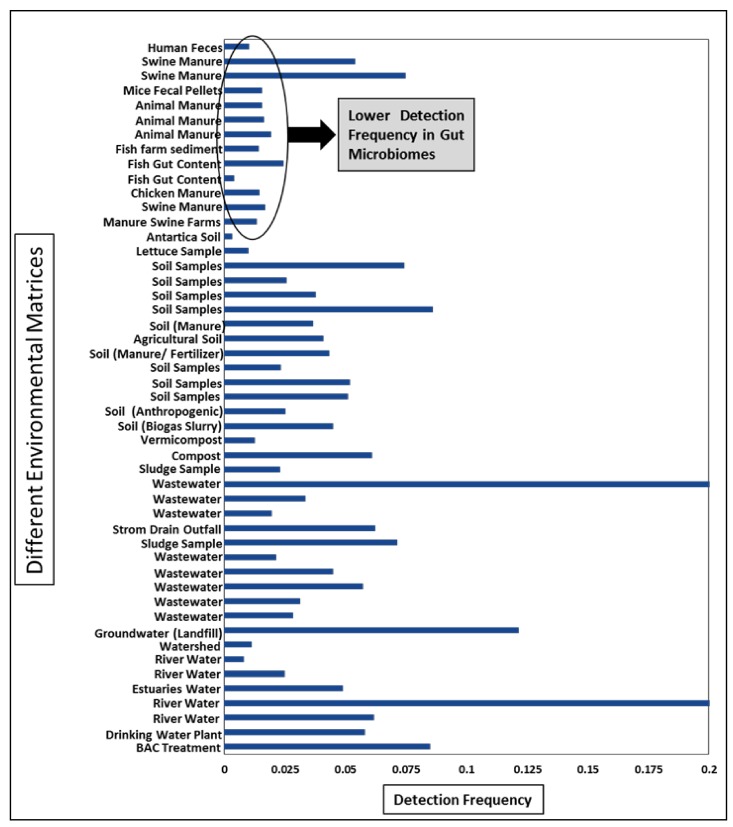
Detection Frequency of different environmental matrices from all included studies.

Only few studies have used and reported diversity indices for depicting the diversity of ARGs [70,71,72]. However, most of the studies have reported no. of unique genes and class wise percentage of detected ARGs for highlighting the diversity of resistance genes. This may lead to wrong interpretation, because the percentage of detected ARG class is dependent on the primers of the genes present on the qPCR array. For example low detection percentage of sulfonamides in many studies [13,17] is because only few primers (5/384) of sulfonamide resistant genes are present in qPCR array [65]. To get an overall idea, we have calculated the detection frequency for comparing the diversity of ARGs between different environmental matrices. Detection frequency here is defined as no. of unique genes detected in a study divided by total number of reactions analyzed. Total number of reactions are obtained by multiplying number of primers used with the number of samples tested in the study. The detection frequency of the ARGs in soils of Antarctica was the lowest among others. In past genomic studies have reported that microorganisms can produce a variety of bioactive antimicrobials in pristine environments [73], therefore, the presence of few ARGs in soils of Antarctica is not surprising. In other environmental matrices, the detection frequency is highly variable, which could be due to demographic [74,75] and other anthropogenic factors [76,77]. An interesting and intriguing trend here is the low detection frequency observed for majority of gut microbiome studies (Figure 4). One possible explanation for this trend could be relatively high bacterial diversity in environmental matrices. For example, Fahrenfeld and Bisceglia have explained that sewer lines can contain more diverse bacteria than fecal samples because resistant bacteria from washed off skin, urine, saliva, sputum and so forth, also go into the waste collection systems along with the fecal microbiome [78]. Chances of horizontal gene transfer are more in diverse bacterial populations [79,80,81]. Hu et al. (2016) has also highlighted, during a comprehensive bacterial mobile resistome study, the existence of potential phylogenetic barrier for horizontal gene transfer of the resistome in bacterial communities [82].

**Figure 5:**
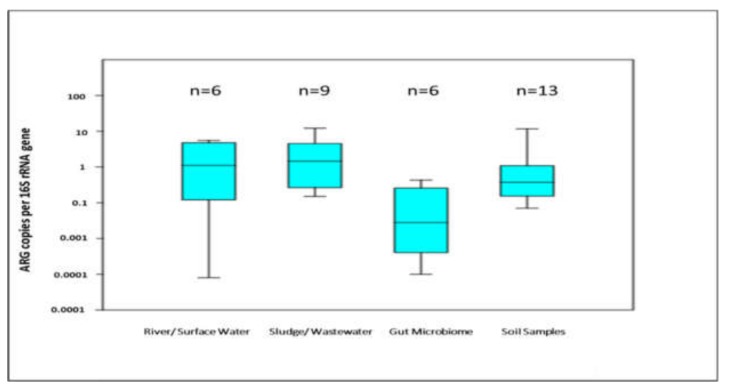
Box and whisker plot depicting the log transformed ARG copies per 16S rRNA gene for different environmental samples, (*n*) represents no. of studies.

This explanation was further strengthened when the average normalized gene abundances (ARGs/16S rRNA gene) of different environmental matrices were compared in our study (Figure 5). The animal manure/ gut microbiome have lower normalized gene abundances than other matrices. Although the difference was not significant when checked by one way ANOVA analysis but the trend of relatively less ARGs/16S rRNA gene was definitely visible. Information derived by detection frequency may give us an idea about the diversity of the genes at the meta-analysis level but calculation and depiction of ARGs diversity via different diversity indices would be the best option to avoid any confusion and misinterpretation.

### Potential in Clinical Laboratories

Although more than 95% of the included studies for ARGs detection and quantification have employed HT-qPCR technology for screening ARGs in various environmental matrices. But this technique also has a great potential and utility in routine clinical laboratories as well. For example a centralized laboratory receiving hundreds of sample per day, where the resistance determination against clinically relevant ARGs is required, can use such an array where limited ARGs can be evaluated against a large sample set. Walker and colleagues have employed a multiplexed nested PCR technique where the 2nd step of the nested PCR was performed on Biomark^TM^ System. An Integrated Fluidic Circuit (Fluidigm) dynamic array capable of analyzing 192 samples against 24 separate PCR assays was used to evaluate 10 antibiotic resistance genes in perianal swab samples collected from patients [83].

## 6. Implications of Data Analysis and Quality Control

Relative abundance of ARGs has so far been expressed in the form of fold change and/or percentage. There are two main equations which have been utilized for analyzing ARGs relative abundance. One or both have been used in almost all reported studies involving HT-qPCR. The first equation (Equation 1) was developed and introduced in 2008 [84]. Scientists are using this method to analyze the fold change of ARGs with respect to a standard and/or control sample. The other equation (Equation 2) was first reported by Looft and colleagues in 2012 while measuring the antibiotic resistance genes in swine gut microbiome [85]. This equation provides an estimated gene copy number and subsequently relative abundance of ARGs with respect to 16S rRNA gene can be calculated.

ΔC_T_ = C_T (ARG)_ − C_T (16S)_, ΔΔCT = ΔC_T (Target)_ − ΔC_T (Reference)_ (1)

Gene Copy Number = 10^(Cut-off − Ct)/(10/3)^ (2)

**Figure 6:**
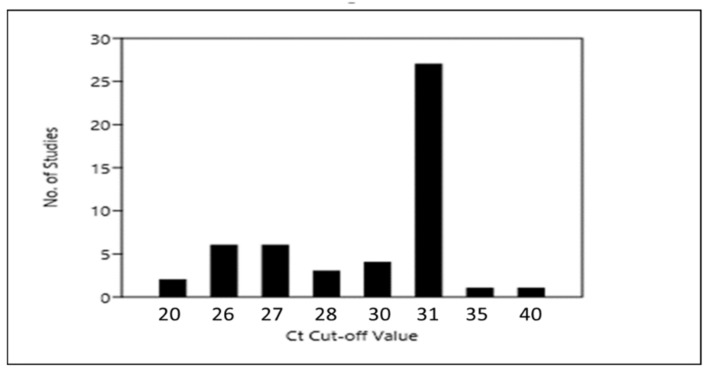
Bar graph showing Ct cut-off values used for the analysis of HT-qPCR data in the included studies.

HT-qPCR has the capability to perform thousands of reactions at once but questions regarding validity of huge number of reactions can be raised because reproducibility and/or quality check of individual reactions become almost impossible. Reliable and reproducible background correction and threshold setting should be used to get a reliable C_T_ value. A C_T_ cut-off value, representing the limit of detection, has to be selected for Equation (2). C_T_ cut-off value can influence the estimated gene copy numbers of the ARGs and 16S rRNA gene. Discrepancies in selection of C_T_ cut-off values are present in various studies, Looft and co-workers in their initial equation has utilized a relatively conservative C_T_ cut-off value of 26 [85]. Different C_T_ cut-off values have been used afterwards, C_T_ cut-off value of as low as 20 [86,87] to as high as 40 [88] have been reported. However in almost half of the studies (25/51) included for the meta-analysis C_T_ cut-off value of 31 has been used (Figure 6). Certain attempts have been made to address these gaps and disparities, for example, recently Stedtfeld and colleagues have compared two C_T_ cut-off values (28 vs. 31) while analyzing ARGs in different environmental and clinical samples and found that higher C_T_ cut-off values can negatively influence the false positive calls. Further C_T_ cut-off value of 31 was found to overestimate the quantity of the ARGs by 10 folds [65].

HT-qPCR gives very precise results, so even a slight inaccuracy in data analysis can easily result in statistically significant but inaccurate and biased findings. Many studies have reportedly measured 16S rRNA on conventional qPCR platform and ARGs on HT-qPCR platform or with different reaction volumes on same platform [45,72,89]. This can raise concerns because instrumental sensitivity and analytical differences are not often considered. If the reference gene (16S rRNA in this case) is not estimated precisely, subsequent normalization can compromise the results of the whole ARGs qPCR array.

There are very few instances where studies were designed to validate and compare the efficiency and quality of HT-qPCR with other techniques. The analytical sensitivity and specificity of HT-qPCR (Applied Biosystems Open Array) and primer design strategies for reducing the extent of validations for developing qPCR arrays for a greater number of targets have been evaluated [90]. Selective validation of the HT-qPCR results by sequencing amplicons have also been reported [91]. Similarly, validation of specificity for selected primers have been checked by analyzing dilutions of extracted DNA from type strains [71] and also from mock communities [65]. Some studies have also tried to compare ARGs abundance by HT-qPCR and metagenomic analysis [85,92]. Recently Sandberg and colleagues have also run standard curves for each of the 48 target genes included in their study. The standard curves represented a dilution series ranging from 10^8^ to 1 ARG copies per reaction for each gene [48]. Similarly, standard curves for ARGs during another study employing HT-qPCR were also reported [47].

The need of the hour is to put a focus on designing and executing such validation studies for evaluating different environmental matrices. Standard curves are needed to be introduced in HT-qPCR based ARGs investigations. Analysis and gene normalization should follow a standard protocol so that the HT-qPCR results coming from different parts of the world are comparable. If 16S rRNA gene is to be measured on conventional platform, the same sample-assay combination should also be evaluated on HT-qPCR platform to minimize the error caused by instrumental variations. MIQE guidelines for publishing research related to qPCR should be implemented in true spirit [93]. Research in this area would be extremely useful in formulating precise strategies aimed at minimizing the public health hazards associated with antibiotic resistance. A comprehensive understanding of the HT-qPCR data analysis warrants concerted efforts from molecular biologists and environmental scientists. This will not only enhance the credibility of the HT-qPCR results but will also produce more uniform, comparable and reliable data.

## 7. Potential of Other Amplification Technologies for ARGs Measurement

The cost of reagents and chemicals is undoubtedly saved in HT-qPCR but the higher cost of HT-qPCR platforms makes it out of the reach of most developing and under developed countries. This is also highlighted by the fact that during the last seven years there is not even a single study from any under developed country (Figure 2). Nucleic acid based point-of-care devices (field-able) can play a critical role in reducing the cost of the platforms [94]. High throughput capacity and automation are possibly some of the key aspects which can be added in these devices. Although a dipstick type of a test is still not possible for ARGs analysis but the need for on-site molecular assessment of ARGs without transporting the samples in a molecular laboratory has already been realized. Scientific community is seeing the exclusion of nucleic acid extraction as a step forward towards on-site ARGs analysis. For example, Kostić and colleagues have reported isothermal amplification of ARGs in 30 min with minimal sample preparation in 384 wells assay cards on a field deployable Gene-Z device [95]. In another isothermal study utilizing MPN-LAMP technique waterborne pathogens were detected in less than 25 min [96]. The use of such techniques in ARGs amplification can bring more simplification in instrumentation further reducing the cost. However there are still many hurdles in the commercialization of such devices possibly due to complexities in primer design and acceptability of amplification methods (except qPCR) in routine diagnostics.

## 8. Conclusions

Our study has provided an effective assessment of the contributions made by HT-qPCR technology in the field of antimicrobial resistance. The drive to introduce more improvements in the existing technology is likely to continue for the foreseeable future as HT-qPCR finds its way to more laboratories and niches. More sophisticated HT-qPCR platforms and/or field deployable devices with higher levels of automation are expected in near future. In addition to reducing the consumable costs particular attention should be given in reducing the instrument/platform costs and technical complexities for the end user. It is anticipated that in future HT-qPCR based resistance studies will continue to have a focus on analyzing linkages among resistance genes with mobile genetic elements and bacterial community structure to determine the health risk in various environmental samples.

## Supplementary Materials

The following are available online.
