# Supplementary material for: Contributions and Challenges of High Throughput qPCR for Determining Antimicrobial Resistance in the Environment: A Critical Review
Source: Molecules. 2019 Jan 3;24(1):163. doi: 10.3390/molecules24010163 (PMC6337382; doi:10.3390/molecules24010163)
Supplement: Supplementary file 1 [file molecules-24-00163-s001.zip › Supplementary Table 1.docx]

| **Database** | **Reference Gene Sequences** | **References** |
| --- | --- | --- |
| ARDB | 23137 | (Liu and Pop, 2009) |
| SARG | 4246 | (Yang et al., 2016) |
| CARD | 2727 | (Jia et al., 2017) |
| Resfinder | >2000 | (Zankari et al., 2012) |
| ARG-ANNOT | 1689 | (Gupta et al., 2014) |
| ARGO | 670 | (Scaria et al., 2005) |
| MEGARes | 3824 | (Lakin et al., 2017) |

**Table S1:** No of ARGs targets in different databases

**References:**

Gupta, S.K., Padmanabhan, B.R., Diene, S.M., Lopez-Rojas, R., Kempf, M., Landraud, L., Rolain, J.-M., 2014. ARG-ANNOT, a new bioinformatic tool to discover antibiotic resistance genes in bacterial genomes. Antimicrob. Agents Chemother. 58, 212–20. doi:10.1128/AAC.01310-13

Jia, B., Raphenya, A.R., Alcock, B., Waglechner, N., Guo, P., Tsang, K.K., Lago, B.A., Dave, B.M., Pereira, S., Sharma, A.N., Doshi, S., Courtot, M., Lo, R., Williams, L.E., Frye, J.G., Elsayegh, T., Sardar, D., Westman, E.L., Pawlowski, A.C., Johnson, T.A., Brinkman, F.S.L., Wright, G.D., McArthur, A.G., 2017. CARD 2017: expansion and model-centric curation of the comprehensive antibiotic resistance database. Nucleic Acids Res. 45, D566–D573. doi:10.1093/nar/gkw1004

Lakin, S.M., Dean, C., Noyes, N.R., Dettenwanger, A., Ross, A.S., Doster, E., Rovira, P., Abdo, Z., Jones, K.L., Ruiz, J., Belk, K.E., Morley, P.S., Boucher, C., 2017. MEGARes: an antimicrobial resistance database for high throughput sequencing. Nucleic Acids Res. 45, D574–D580. doi:10.1093/nar/gkw1009

Liu, B., Pop, M., 2009. ARDB--Antibiotic Resistance Genes Database. Nucleic Acids Res. 37, D443-7. doi:10.1093/nar/gkn656

Scaria, J., Chandramouli, U., Verma, S.K., 2005. Antibiotic Resistance Genes Online (ARGO): a Database on vancomycin and beta-lactam resistance genes. Bioinformation 1, 5–7.

Yang, Y., Jiang, X., Chai, B., Ma, L., Li, B., Zhang, A., Cole, J.R., Tiedje, J.M., Zhang, T., 2016. ARGs-OAP: online analysis pipeline for antibiotic resistance genes detection from metagenomic data using an integrated structured ARG-database. Bioinformatics 32, 2346–2351. doi:10.1093/bioinformatics/btw136

Zankari, E., Hasman, H., Cosentino, S., Vestergaard, M., Rasmussen, S., Lund, O., Aarestrup, F.M., Larsen, M. V., 2012. Identification of acquired antimicrobial resistance genes. J. Antimicrob. Chemother. 67, 2640–2644. doi:10.1093/jac/dks261
